# A study on predicting the length of hospital stay for Chinese patients with ischemic stroke based on the XGBoost algorithm

**DOI:** 10.1186/s12911-023-02140-4

**Published:** 2023-03-22

**Authors:** Rui Chen, Shengfa Zhang, Jie Li, Dongwei Guo, Weijun Zhang, Xiaoying Wang, Donghua Tian, Zhiyong Qu, Xiaohua Wang

**Affiliations:** 1 Refined Management Office, Cangzhou Central Hospital, Cangzhou, China; 2 National Population Heath Data Center, Chinese Academy of Medical Sciences and Peking Union Medical College, Beijing, China; 3 School of Economics and Management, Hebei University of Technology, Tianjin, China; 4 School of Social Development and Public Policy, Beijing Normal University, Beijing, China

**Keywords:** Ischemic stroke, XGBoost algorithm, Length of hospital stay (LOS), Machine learning (ML) model

## Abstract

**Background:**

The incidence of stroke is a challenge in China, as stroke imposes a heavy burden on families, national health services, social services, and the economy. The length of hospital stay (LOS) is an essential indicator of utilization of medical services and is usually used to assess the efficiency of hospital management and patient quality of care. This study established a prediction model based on a machine learning algorithm to predict ischemic stroke patients’ LOS.

**Methods:**

A total of 18,195 ischemic stroke patients’ electronic medical records and 28 attributes were extracted from electronic medical records in a large comprehensive hospital in China. The prediction of LOS was regarded as a multi classification problem, and LOS was divided into three categories: 1–7 days, 8–14 days and more than 14 days. After preprocessing the data and feature selection, the XGBoost algorithm was used to build a machine learning model. Ten fold cross-validation was used for model validation. The accuracy (ACC), recall rate (RE) and F1 measure were used to evaluate the performance of the prediction model of LOS of ischemic stroke patients. Finally, the XGBoost algorithm was used to identify and remove irrelevant features by ranking all attributes based on feature importance.

**Results:**

Compared with the naive Bayesian algorithm, logistic region algorithm, decision tree classifier algorithm and ADaBoost classifier algorithm, the XGBoot algorithm has higher ACC, RE and F1 measure. The average ACC, RE and F1 measure were 0.89, 0.89 and 0.89 under the 10-fold cross-validation. According to the analysis of the importance of features, the LOS of ischemic stroke patients was affected by demographic characteristics, past medical history, admission examination features, and operation characteristics. Finally, the features in terms of hemiplegia aphasia, MRS, NIHSS, TIA, Operation or not, coma index etc. were found to be the top features in importance in predicting the LOS of ischemic stroke patients.

**Conclusions:**

The XGBoost algorithm was an appropriate machine learning method for predicting the LOS of patients with ischemic stroke. Based on the prediction model, an intelligent medical management prediction system could be developed to predict the LOS based on ischemic stroke patients’ electronic medical records.

**Supplementary Information:**

The online version contains supplementary material available at 10.1186/s12911-023-02140-4

## Background

Stroke, as the second most common cause of death worldwide and the leading cause of acquired disability in adults, was a kind of acute cerebrovascular disease characterized by the focal neurological deficit, mainly including ischemic stroke and hemorrhagic stroke [1, 2]. Stroke had become the second most fatal disease in the world and its disability-adjusted life years (DALYs) and the years of life lost (YLLs) due to stroke ranked third globally [3, 4]. China faced the biggest stroke challenge from stroke in the world. In China, cerebrovascular disease’s mortality rate was 149.49/100,000, accounting for 1.57 million deaths in 2018 [5]. The 2016 global burden of disease (GBD) study estimated that the estimated lifetime risk of stroke in China from 25 was the highest, as high as 39.3% [4].

Ischemic stroke, which is the most common stroke type and accounts for 70%-80% of strokes, was caused by cerebral artery occlusion [6]. In China, the incidence rate of ischemic stroke has been rising, especially for young people under 45 years old and it has brought a heavy burden to the family, national health, social services, and economy [7, 8]. The increasing trend of ischemic stroke incidence rate was young patients, rather than the elderly, which had caused a heavier social and economic burden [9]. In 2013, the results of the particular investigation of cerebrovascular disease epidemiology showed that The weighted incidence rate of ischemic stroke was 181.7/100,000 in males and 151.9/100,000 in females [10]. Although the management of ischemic stroke had improved over time, it came with a high economic burden. In 2017, the prevalence of ischemic stroke was 1981/100,000, the disability adjusted life year (DALY) is 1007/100,000 and the incidence rate of ischemic stroke in China rose from 112/100,000 in 2005 to 156/100,000 in 2017 [11]. According to the data, in 2017, the average hospitalization expenses of ischemic stroke patients in China were 18525 yuan, with an increase of 60% compared with 2007 and the average annual growth rate of ischemic stroke far exceeded the growth rate of GDP [11].

More than half of the costs associated with ischemic stroke were related to hospitalization. Notably, one of the best predictive markers for hospitalization expenses among stroke patients was length of hospital stay (LOS). In China, many tertiary hospitals have begun to implement the single disease payment system. Ischemic stroke is one of the diseases that need to be paid according to the single disease payment system. The LOS and hospitalization cost played a vital role in the clinical pathway and hospitalization cost accounting. Therefore, it was reasonable to focus on sustainable quality improvement efforts to reduce LOS. Accurate prediction of LOS had become increasingly important for health care systems, and reducing the LOS had the potential for considerable savings in the public hospital system. Previous studies showed that LOS was associated with stroke severity, complications during the hospital stay, stroke subtype, course of diseases, treatment, hypertension, diabetes mellitus, and smoking, age etc. At present, studies mainly focused on the analysis of influencing factors of hospitalization costs for ischemic stroke, and further studies on the prediction of LOS were relatively few [12, 13]. The existing studies were mainly based on traditional statistical analysis methods, and the research starting from data mining methods lagged behind [14]. However, with intelligent medical technology development, data mining has been widely used in the medical field due to its advantages of efficiently extracting value information from massive medical data. Therefore, this study aimed to establish a prediction model based on the machine learning algorithm to predict ischemic stroke patients’ LOS.

The benefits of measuring hospital service level were economical and extended to other medical care aspects, including patients themselves. First, predicting LOS enabled hospitals to identify patients with a high risk of long-term hospitalization and then use the optimized treatment plan to treat them or carry out early intervention to prevent other complications. Second, predicting LOS could lead to earlier discharge to the home, or a cheaper medical institution supported by community nurses and doctors. Specifically, as an important index to measure inpatients’ resource consumption, accurately predicting patients’ service level provides a better management means for the hospital to improve the utilization rate of resources. Therefore, predicting patients’ LOS could help medical service institutions enhance patient satisfaction, make more effective use of human resources and facilities, reduce treatment costs and improve sustainability.

In recent years, a major contributor to realizing these improvements in health care outcomes had been the increasing use of big data analytics. Previous studies employed data analytic approaches, such as Regression methods, Naive Bayes, multi-layer neural network (MLP), K-Nearest Neighbors (KNN) algorithm, classification and regression tree (CART) and support vector regression, to develop a model for the early prediction of patients’ LOS [15–17]. The objective of this study was to describe the development and validation of a new machine learning model to predict the LOS of patients at the time of admission.

The Extreme Gradient Boosting (XGBoost) algorithm, a machine learning algorithm, could improve the integration of multiple decision trees by using the gradient promotion method, which has the characteristics of high accuracy, difficulty in overfitting and scalability. The XGBoost could also deal with high-dimensional sparse features in a distributed manner. At present, the XGBoost algorithm had been widely used in fields such as short-term passenger demand, fault monitoring and commercial default prediction [18–20]. In recent years, the XGBoost algorithm had been gradually applied in the field of health care and had been involved in the prediction of fraud risk, heart disease, pneumonia, diabetes and cardiovascular disease [21, 22]. Therefore, it was feasible and practical to apply the XGBoost algorithm to predict of hospitalization days of ischemic stroke patients.

This study aims to establish an integrated prediction model based on the XGBoost algorithm to predict the LOS of ischemic stroke patients to provide better decision support for hospital management of medical resources and doctors’ choice of the treatment plan, thus saving the treatment costs of patients in the hospital and improving the later rehabilitation effect.

## Methods

**Fig. 1:**
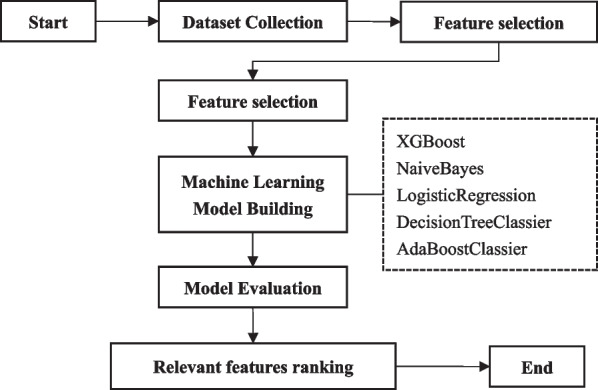
The steps of building the machine learning model to predict the LOS of ischemic stroke patients

Several steps were used in this study, as showed in Fig. 1.

### Step1: The dataset collection

The dataset of this study included adult patients who were admitted between 2017 and 2018 in the Cangzhou Central Hospital in Hebei Province, China. A total of 18,195 cardiac patients records and 28 attributes were extracted from electronic medical records (Shown in Additional file 1). These attributes were collected prospectively by experienced physicians and trained nurses. The attributes of the dataset included demographic characteristics, past medical history, admission examination characteristics and operation characteristics. Admission diagnosis and the primary treating physician were reviewed to verify selection criteria. This study included all patients who were admitted to the cardiac wards under the clinical cardiology service. Patients who were admitted under other services in the cardiac wards for non-cardiac-related admitting diagnosis were excluded. The LOS was determined by subtracting the discharge date from the admission date. In this study, the prediction of LOS was regarded as a multi classification problem. According to the medical research, the length of hospitalization for ischemic stroke in the range of 7–14 days was a protective factor for the adverse outcome after discharge. Meanwhile, in combination with the provisions of China’s medical insurance policy, the length of hospitalization for ischemic stroke in the range of 1–7 days, 8–14 days, and more than 14 days was discretized for prediction, and the data ratio of the three types was 1.7:3.9:1.

### Step2: Feature selection

Firstly, some variables were screened according to the literature, and then the variables finally included in the model were by random forest method. Finally, 28 characteristic variables and label variables of length of stay were extracted. By eliminating the invalid variables, missing value filling and data integration, 18,195 training samples of ischemic stroke patients were collected, forming a 28$\times$18195 feature-sample training dataset matrix. The 28 characteristic variables could be divided into demographic characteristics, past medical history, admission examination information and operation features, and the specific features were shown in Table 1.

**Table 1:** Variable of the dataset

Categories	Variables
Demographic characteristics (5)	Sex, age, marriage status, occupation, hospital payment method
Physical measurement (1)	BMI
Past medical history (10)	Smoking history, Transient ischemic attack (TIA), hypertension, hyperlipidemia, diabetes, coronary heart disease, peripheral arterial disease, atrial fibrillation, hyper homocysteinemia, epilepsy
Characteristics of admission examinatio n(10)	Admission Modified Rankin Scale (MRS), admission National Institute of Health Stroke Scale (NIHSS), coma index, cognitive impairment, hemiplegia aphasia, trauma, respiratory infection, dizziness, critical or not, admission path
Operation features (2)	Operation or not, postoperative sequelae

The LOS for each inpatient admission was calculated by subtracting the date and time of admission from the date of discharge. The time elapsed between the previous inpatient admission and the current index admission was calculated by the date difference between them.

### Step3: Data pre-processing

Since the data of ischemic stroke patients were manually entered, there might be missing values, abnormal values, repeated values, etc. These abnormal data affected the real information in the model learning data and had adverse effects on the final performance of the model. Therefore, the data should be preprocessed before model training. A few data pre-processing steps were taken before analyzing the data:

1. All records of the same patient were merged if the patient had multiple hospitalizations on the same day to the same medical unit. That applied to both medical and surgical inpatients.
2. Admissions that were recorded as having occurred within 24 h of the immediately prior index discharge were excluded from the admission set.
3. Missing value referred to the missing data caused by data loss in the process of data entry or extraction. There were two ways to deal with the missing value. For the features with more missing values, the deletion operation was usually used, and the features with less missing values could be filled by means of means, modes, median and other methods. In this study, the missing values in the data of patients with ischemic stroke were less, so the mean value was calculated by the row fill method.
4. Abnormal values indicated that the data in a feature was far beyond the feature’s overall distribution. In practice, it might be caused by an error in the input, and the abnormal value might have a significant impact on the prediction results. When the dimensions and orders of magnitude of different features were different, using the original data would make the algorithm biased to additional features, affecting the prediction performance of the model. Therefore, this study used standard normalization to standardize the data.
5. Inconsistent and/or erroneous components, such as age discrepancies, or a discharge date that preceded the admission date, were excluded.
6. The 10-fold cross-validation was used to train the model. The dataset was separated randomly into a training/validation set of 14,556 admissions (80%), and a test set of 3639 admissions (20%).

#### Step4: Model building

Previous studies found that the XGBoost algorithm not only has a high prediction accuracy, but also has a high efficiency when the amount of data is relatively large. Therefore, this study used XGBoost algorithm to build the LOS prediction model.

### The XGBoost algorithm

The XGBoost algorithm was based on the CART classification and regression tree. It generated a basic learning device based on the selection of sample features, then fits the residuals by segments, and finally forms a comprehensive prediction model. A single CART tree could train the corresponding prediction score of $F(X_i)$ for each sample. The XGBoost algorithm was an additive model obtained by integrating the prediction results of multiple carts, as shown in Equation (1).

1 $$\begin{aligned} \hat{y}_{l}=\phi \left( X_{i}\right) =\sum _{k=1}^{K} f_{k}\left( X_{i}\right) , \quad f_{k} \in F \end{aligned}$$

$X_i$ represents the *i*th hospitalized patient with ischemic stroke. $\hat{y}_{l}$ represents the predicted results of $X_i$. *K* represents all cart tree based learners and *F* is the space of the CART tree. According to Equation (1), the XGBoost algorithm maps it to the corresponding leaf node by applying K CART decision rules, and adds up the mapping scores of each leaf node to obtain the final classification prediction score of the sample.

Meanwhile, this study applied the NaiveBayes, Logistic Regression, DecisionTreeClassier and AdaBoostClassier algorithm to establish the LOS prediction models, and compared them with the model built by the XGBoost algorithm to test and verify the performance of the model.

### Main steps of model building

The main steps of model building were as follows:

1. The dataset was separated randomly into a training/validation set and a test set;
2. The training/validation $Dx_i$ was divided into 10 mutually exclusive subsets with similar size, and the data distribution in each subgroup was as consistent as possible, thus 10 sample subsets $Dx_{ij} (J = 1,2,\ldots )$ were obtained;
3. For the *j*-th subset $Dx_{ij}$, it was used as the validation set, and the union of the other nine sample subsets was used as the training set, and the XGBoost algorithm and other four algorithms were used to train the model;
4. Repeated the third step and 10 sample subsets were used for training in turn, thus we could obtaining five base prediction models $Mi (I = 1,2)$. The average value of the performance measures of the five predicting models was used as the performance index of the prediction model of LOS. The grid search of sklearn-Gridsearchcv in Python was used to systematically traverse multiple parameter combinations and determine the best parameters through cross validation determine the parameters in the model. Finally, the final prediction model of LOS was obtained.
5. Use the test set to evaluate the generalization ability of the final predicting model.

### Main parameters of model building

The main parameters settings were shown in Table 2.

**Table 2:** Main parameters of the model for predicting LOS of ischemic stroke patients

Parameters	Value
booster	gbtree
objective	multi: softmax
n_estimators	1000
max_depth	3
min_child_weight	3
learning_rate	0.2
gamma	0
subsample	0.8
colsample_bytree	0.8

### Cross validation

Cross-validation was a technique to evaluate predictive models by partitioning the original sample into a training set to train the model, and a test set to evaluate it. In the study, the steps were as follows: (1) Divide the dataset into training/validation set (0.8) and test set(0.2), and put the test set aside. (2) Of the 10 subsamples, nine parts used for training the model, and the rest 1 part was used as the validation set. (3) The cross-validation process was then repeated 10 times, with each of the 10 subsamples used exactly once as the validation set. (4) The 10 results from the folds then be averaged (or otherwise combined) to produce a single estimation. (5) After 10 training sessions, we had 10 different models. (6) Evaluate the effects of 10 models and selected the hyperparameter that worked best. (7) The optimal hyperparameters were used and the final model was obtained. (8) The testing dataset were used as test sets to evaluate the model.

### Step5: Model evaluation

In this paper, combined with medical research and China’s medical insurance policy, it is a typical three classification problem to convert the length of stay of ischemic stroke into discrete variables of three stages. The definition of prediction results $(i = 1,2,3; j = 1,2,3)$ represents the number of samples predicted from class *i* to class *j*, where *I* represent the real value category of the sample, and *j* represents the category of the predicted value of the sample. In this paper, the accuracy (ACC), recall rate (RE) and $F_1$ measure were used to evaluate the performance of the prediction model of LOS of ischemic stroke patients.

2 $$\begin{aligned} A C C= & {} \sum _{i=j=1}^{3} P_{i j} / \sum _{i, j=1}^{3} P_{i j} \end{aligned}$$

3 $$\begin{aligned} R E= & {} \frac{1}{3} \sum _{i=j=1}^{3} P_{i j} / \sum _{j=1}^{3} P_{i j} \end{aligned}$$

4 $$\begin{aligned} F_{1}= & {} 2 * R E * P R E /(R E+P R E) \end{aligned}$$

### Step6: Relevant features ranking

The information gain method was used to determine the most important features, which can significantly affect the accuracy of the model. The technique could sort all attributes according to their importance and identify and remove irrelevant features.

## Results

### The baseline characteristics of patients with ischemic stroke among different LOS groups

A total of (18,195) cardiac visits for patients with ischemic stroke who were admitted to the cardiac center from 2017 and 2018 were analyzed. Of these, the baseline features were shown in Table 3.

**Table 3:** Baseline features of the dataset

Variables	Value	Length of stay (LOS)			P-value
		1–7 days (n = 4133)	8–14 days (n = 11624)	More than 14 days (n = 2402)	
BMI	–	22.76	23.57	24.39	.000***
Age	–	56	60	62	.000***
Sex	Male	2127	6112	1566	.000***
	Female	2006	5512	836	
Marriage status	Married	4015	11316	2345	.004**
	Unmarried	89	184	25	
	Divorced or widowed	29	124	32	
Occupation	Civil servant	54	90	31	.000***
	Personnel of enterprises and institutions	236	471	84	
	Worker	139	360	77	
	Farmer	2957	7592	1617	
	Retiree	448	2141	419	
	Student	37	74	4	
	Individual household	33	115	17	
	Others	229	781	153	
Hospital payment method	Basic medical insurance for urban employees	849	3073	609	.000***
	Health insurance for urban and rural residents	2965	7757	1628	
	Commercial medical insurance	292	732	151	
	Others	27	62	14	
	Basic medical insurance for urban employees	849	3073	609	
	Health insurance for urbanand rural residents	2965	7757	1628	
	Commercial medical insurance	292	732	151	
Smoking history	No	2837	8066	1457	.000***
	Yes	1296	3558	945	
Transient ischemic attack (TIA)	No	1661	6675	2204	.000***
	Yes	2472	4949	198	
Hypertension	No	2192	4319	702	.000***
	Yes	1941	7305	1700	
Hyperlipidemia	No	3419	9562	2053	.001**
	Yes	714	2062	349	
Diabetes	No	3662	9386	1658	.000***
	Yes	471	2238	744	
Coronary heart disease	No	3605	9557	1785	.000***
	Yes	528	2067	617	
Peripheral arterial disease	No	3097	7570	1353	.000***
	Yes	1036	4054	1049	
Atrial fibrillation	No	4107	11468	2315	.000***
	Yes	26	156	87	
Hyper homocysteinemia	No	3708	10171	2136	.000***
	Yes	425	1453	266	
Epilepsy	No	4028	11480	2337	.000***
	Yes	105	144	65	
Admission MRS	–	1	1	2	.000***
Admission NIHSS	–	9.20	12.21	15.23	.000***
Coma index	–	15	15	15	> 0.05
Cognitive impairment	No	4078	11567	2392	.000***
	Yes	55	57	10	
Hemiplegia aphasia	No	4128	11616	2302	.000***
	Yes	5	8	100	
Trauma	No	4109	11592	2376	.000***
	Yes	24	32	26	
Respiratory infection	No	3899	10817	2061	.000***
	Yes	234	807	341	
Dizziness	No	3678	11172	2374	.000***
	Yes	455	452	28	
Whether it critical or not	No	4108	11595	2336	.000***
	Yes	25	29	66	
Admission path	Outpatient	3760	10387	2022	.000***
	Hospital transfer	370	1230	377	
Operation or not	No	4122	11567	2215	.000***
	Yes	11	57	187	
Postoperative sequelae	No	3712	9981	1979	.000***
	Yes	421	1643	423	

**P < 0.01; ***P < 0.001

### The performance of the different machine learning models evaluated

In this study, the XGBoost algorithm, Naive Bayes algorithm, logistic regression algorithm, decision tree classifier and AdaBoost classifier were used to build the model. As the LOS in this study was a three classification variable, this study used one verse rest (OVR) strategy to establish the model by constructing three binary classifiers. During training, the samples of one category were classified into one category and the samples of other categories were classified into another category. In this way, for the samples of an unknown category, the three classifiers would have a decision score (probability), and then took the category with the highest decision score as the category of the sample. The datasets of patients with ischemic stroke were predicted 10 times on the prediction model to verify the stability of the model. The three evaluation indexes of each prediction results were shown in Table 4.

**Table 4:** Comparison of the performance of different models

	ACC	RE	F_1_
XGBoost	0.89	0.89	0.89
NaiveBayes	0.63	0.65	0.60
LogisticRegression	0.74	0.75	0.74
DecisionTreeClassifier	0.85	0.85	0.85
AdaBoostClassifier	0.83	0.83	0.83

It can be found that the XGBoost algorithm has the best effect in predicting the length of stay. The ACC was 0.89, which indicates that the model had accurate prediction performance for LOS in each stage. The RE reached 0.89, which indicates that the model could better identify the hospitalization time in each stage in the data. The F1 measure was 0.89, indicating that the model still had good performance when the comprehensive recall rate and accuracy rate were used. Decision tree classifier and AdaBoost classifier also have high prediction accuracy and recall, but they are slightly worse than the XGBoost algorithm. The results showed that xgboost algorithm has better prediction effect.

The three evaluation indexes of the XGBoost prediction model were shown in Table 5. The standard deviations of ACC, RE and $F_1$ measures were all less than 0.05, which indicates that the model has good stability. In conclusion, the integrated model based on the XGBoost algorithm could effectively predict the hospitalization days of ischemic stroke patients and provide positive decision support for patients and doctors.

**Table 5:** The performance of the XGBoost model evaluated using 10-fold cross-validation method

	ACC	RE	$F_1$
Mean	0.89	0.89	0.89
SD	0.02	0.04	0.03
P-value	.022*	.045*	.034*

*P < 0.05

### The importance of predictive characteristics of hospitalization days in patients with Ischemic stroke

The XGBoost algorithm was used to rank the importance of the features in the prediction of hospitalization days of patients with Ischemic stroke, and the number of times that the features were used as segmentation samples in all cart trees was taken as the importance index. The features including NIHSS, TIA, Operation or not, hemiplegia aphasia, MRS, coma index, critical or not critical, occupation, dizziness, and respiratory infection were found to be the top ten features in importance in predicting the LOS of ischemic stroke patients. The results are shown in Table 6.

**Table 6:** The importance of features of prediction model

Ranking	Variables	Descriptions	Feature importance
1	Hemiplegia aphasia	Did the patient have symptoms of partial aphasia on admission	0.486
2	MRS	Ability of life (MRS) score at admission	0.305
3	NIHSS	NIHSS score at admission	0.221
4	TIA	Whether the patient had transient ischemic attack (TIA symptoms) on admission	0.181
5	Operation or not	Is the patient treated surgically	0.113
6	Coma index	Coma index	0.031
7	Critical or not	Was the patient critically ill at admission	0.027
8	Occupation	Different occupation types	0.021
9	Dizziness	Dizziness or not	0.018
10	Respiratory infection	Respiratory tract infection or not	0.017

The result showed that the four features of hemiplegia aphasia, MRS at admission, admission NIHSS value and TIA had the most significant impact on predicting LOS of ischemic stroke patients. Other clinical manifestations such as Operation or not, coma index, critical or not, dizziness and respiratory infection were also important factors affecting LOS. Although the patients’ demographic characteristics, such as age, BMI, marital status and occupation also had a significant impact on LOS, only occupation had a greater impact. Overall, the aspects of demographic features, past medical history, admission examination characteristics, and operation characteristics had a significant influence on the LOS of patients with ischemic stroke. The features of admission examination and operation greatly influenced the hospitalization time of patients with ischemic stroke, which showed that the LOS in patients with ischemic stroke was a comprehensive problem mainly affected by various factors.

## Discussion

This study aimed to develop a new machine learning model to predict the LOS of ischemic stroke patients at admission. The results showed that compared with the naive Bayesian algorithm, logistic region algorithm, decision tree classifier algorithm and ADaBoost classifier algorithm, the machine learning model built with the XGBoot algorithm was better in ACC, RE and F1 measure. The prediction model developed using the XGBoost algorithm have high prediction accuracy and good stability in predicting LOS in Chinese patients with ischemic stroke. Thus, the XGBoost algorithm was an appropriate machine learning method for predicting LOS of ischemic stroke patients.

In recent years, researchers had carried out a lot of studies on analyzing disease risk factors, diagnostic criteria and treatment effects using machine learning technology based on structured data in electronic medical records [23]. The results showed that the essential attributes that have the most decisive influence on the LOS of ischemic stroke patients were: hemiplegia aphasia, MRS at admission, admission NIHSS value, TIA, coma index, critical or not, occupation, dizziness and respiratory infection. It has been found factors such as diabetes, atrial fibrillation and hypertension were essential factors affecting the LOS of ischemic stroke patients [15, 24]. Compared with previous studies, the impact of Hemiplegia aphasia, MRS at admission and admission NIHSS value on the LOS of ischemic stroke patients was confirmed for the first time in this study. Although many studies had used machine learning models to predict LOS of ischemic stroke, the prediction model proposed in this study had better performance and could effectively predict LOS of patients with ischemic stroke.

During the treatment of ischemic stroke, it was helpful for doctors to make better treatment plans to find the essential factors that affect the LOS [25]. It could also help hospital managers better arrange and dispatch hospital beds, drugs and nursing staff, and help hospitals better improve hospital management efficiency [26]. At the same time, it could provide accurate hospitalization information for patients and their families, so that they could better arrange hospitalization expenses, life care and other aspects.

Although the existed prediction models based on data mining algorithms could predict the length of hospitalization to a certain extent, the consistency of the prediction variables of each model was not high, due to the complexity and variability of the influencing factors of LOS [16, 17, 22]. The main reasons for this were as follows:(1) Although some studies had collected electronic medical record data, the standards of electronic medical record were not uniform, and the data variables were quite different; (2) Each research focused on different perspectives, and researchers choose data variables based on a certain perspective, leading to selection bias.

Before selecting variables, a literature review was conducted to sort out the main factors affecting LOS found in previous studies firstly [16, 17]. Then, the variables finally included in the model by using random forest method and 28 variables were included finaly. Although 28 variables accounted for a small proportion in the total number of variables in the electronic medical records, the prediction model established in the study had a high accuracy, which could help to predict LOS using the forward-looking data collected at admission. Therefore, the model established in this study was simple and accurate, which is more in line with the actual needs of hospital managers and doctors to improve service quality and efficiency, and could help to improve the life quality of patients and their families during hospitalization.

However, there were several limitations in the study that need to be recognized. First, the population included in this study covers one prefecture level city in northern China which might negatively impact the performance of the model. In contrast, data from other cities or applying the model in other health organizations might have a different result. Second, only two years of patient records were collected in the study. The external environmental changes in the last two years (such as medical insurance payments, public hospital reform systems, etc.) might significantly impact the LOS of patients and, to a certain extent, affect the accuracy of the model.

## Conclusion

The XGBoost algorithm was an appropriate machine learning method for predicting the LOS of patients with ischemic stroke. In the ML model on predicting the LOS of ischemic stroke patients, demographic characteristics, past medical history, admission examination characteristics, and operation characteristics significantly impacted hospitalization days. Based on the prediction model, an intelligent medical management prediction system could be developed to predict the LOS based on electronic medical records of ischemic stroke patients.

## Supplementary Information

**Additional file 1**: The dataset of the study.

## Data Availability

Due to privacy laws and the data user agreement among the Cangzhou Central Hospital, Hebei University of Technology and Beijing Normal University, the dataset used and/or analyzed during the current study were anonymously processed. The anonymous data had been authorized for use by the Cangzhou Central Hospital and could be used for scientific research. The sample data has been authorized by Cangzhou Central Hospital to be used as public data for researchers without any additional permission. Full data used in the study has been stored in the research team’s database. If researchers need to use the full data, they could contact the corresponding author to obtain the research data after re-signing a license agreement with Cangzhou Central Hospital.

## Abbreviations

LOS: Length of hospital stay
ML: Machine learning
ACC: Accuracy
RE: Recall rate
XGBoost: Extreme gradient boosting
TIA: Transient ischemic attack
MRS: Modified Rankin Scale
NIHSS: National Institute of Health Stroke Scale
